# A multi-center naturalistic study of a newly designed 12-sessions group psychoeducation program for patients with bipolar disorder and their caregivers

**DOI:** 10.1186/s40345-020-00190-5

**Published:** 2020-09-01

**Authors:** Susan Zyto, Nienke Jabben, Peter F. J. Schulte, Eline J. Regeer, Peter J. J. Goossens, Ralph W. Kupka, the Task Force Psychotherapy of the Dutch Foundation for Bipolar Disorders

**Affiliations:** 1 Mental Health Service Organisation North Holland North, Center for Psychosomatic Medicine, Hoorn, The Netherlands; 2 Department of Psychiatry, Zuyderland Medical Center, Sittard, The Netherlands; 3 Mental Health Service Organisation North Holland North, Alkmaar, The Netherlands; 4 Altrecht Institute for Mental Health Care, Utrecht, The Netherlands; 5 Dimence Group Mental Health Care Center, Deventer, The Netherlands; 6 University Center for Nursing and Midwifery, Department of Public Health and Primary Care, Faculty of Medicine and Health Sciences, Ghent University, Ghent, Belgium; 7 Department of Psychiatry, Amsterdam University Medical Center, Vrije Universiteit, Amsterdam Public Health Research Institute, Amsterdam, The Netherlands; 8 GGZinGeest Center for Mental Health Care, Amsterdam, The Netherlands

**Keywords:** Bipolar disorder, Psychoeducation, Self-management, Expressed emotions, Caregivers

## Abstract

**Background:**

Psychoeducation (PE) for bipolar disorder (BD) has a first-line recommendation for the maintenance treatment phase of BD. Formats vary greatly in the number of sessions, whether offered individually or in a group, and with or without caregivers attending. Due to a large variation in formats in the Netherlands, a new program was developed and implemented in 17 outpatient clinics throughout the country. The current study investigated the feasibility of a newly developed 12-sessions PE group program for patients with BD and their caregivers in routine outpatient practice and additionally explored its effectiveness.

**Methods:**

Participants in the study were 108 patients diagnosed with BD, 88 caregivers and 35 course leaders. Feasibility and acceptance of the program were investigated by measures of attendance, and evaluative questionnaires after session 12. Preliminary treatment effects were investigated by pre- and post-measures on mood symptoms, attitudes towards BD and its treatment, levels of self-management, and levels of expressed emotion.

**Results:**

There was a high degree of satisfaction with the current program as reported by patients, caregivers, and course leaders. The average attendance was high and 83% of the patients and 75% of the caregivers completed the program. Analyses of treatment effects suggest positive effects on depressive symptoms and self-management in patients, and lower EE as experienced by caregivers.

**Conclusions:**

This compact 12-sessions psychoeducation group program showed good feasibility and was well accepted by patients, caregivers, and course leaders. Preliminary effects on measures of self-management, expressed emotions, and depressive symptoms were promising. After its introduction it has been widely implemented in mental health institutions throughout the Netherlands.

## Background

Bipolar disorder (BD) is characterised by recurrent depressive, hypomanic, manic, and mixed mood episodes (American Psychiatric Association 2013). Treatment guidelines recommend prophylactic maintenance pharmacotherapy as a first line treatment (Yatham et al. 2018). However, medication adherence is typically poor (Arvilommi et al. 2014) and even despite adequate pharmacotherapy many patients relapse or suffer from persisting residual symptoms (Gitlin et al. 1995). Psychoeducation (PE) is regarded as an effective adjunct to medication (Conolly et al. 2011) and has a first-line recommendation for the maintenance treatment phase of BD (Yatham et al. 2018).

PE aims at improving insight into the illness, medication adherence, awareness of triggers, identification of prodromal symptoms of recurrence, and offering coping strategies to prevent mood relapse in order to empower patients and to encourage them to be active participants in treatment (Miklowitz 2009). PE is offered to patients and/or their caregivers in the euthymic phase of the illness next to pharmacotherapy. Most randomized controlled trials focus on group and caregiver PE (Soo 2018). In these studies, PE was offered in various formats. In the largest RCT to date, a 21-session PE group program for patients was associated with a significant longer time to recurrence of depressive, manic, hypomanic, and mixed episodes, fewer recurrences, and a lower number of hospitalizations and hospitalization days with favourable long-term effects up to 5 years (Colom et al. 2003a; Colom et al. 2009). This was replicated in a second RCT (Colom et al. 2003b). A pilot study with a 12-session PE group program found a positive trend in reduction of recurrences in the intervention group (Castle et al. 2007). A subsequent RCT showed fewer depressive and manic recurrences in the treatment group (Castle et al. 2010). Eight sessions of group PE delivered during 2 weeks were superior to the same number of free individual discussions with a nurse with regard to recurrence and hospitalization (Chen et al. 2019). Two trials reported no significant difference in recurrences between a control group and group PE of 16 biweekly sessions (de Barros Pellegrinelli et al. 2013) or 21 weekly sessions (Morriss et al. 2016), possibly due to a preponderance of participants with a high number of previous mood episodes. Kallestad et al. (2016) found that 10 weekly sessions of group PE followed by eight booster-sessions over the next 2 years were superior to three sessions of individual PE in delaying next hospitalization. Parikh et al. (2013) compared 6 weekly group PE sessions with 20 individual CGT sessions and found no difference between the two groups in weekly mood ratings nor in recurrence rates over the next 18 months (Parikh et al. 2013).

In a study of caregivers receiving twelve 90-mins sessions of group PE focusing on increasing knowledge of bipolar disorder and training in coping skills, the corresponding patients had less recurrences during the subsequent year (Reinares et al. 2008). Madigan et al. (2012) found better quality of life after one and 2 years in patients whose caregivers had participated in 5 weekly 2 h sessions of psychoeducation in comparison to treatment as usual. Only one study investigated the combined attendance of euthymic patients and caregivers in a PE course. In this RCT of patients and their caregivers receiving 12 weekly 90 min PE sessions, D’Souza et al. (2010) found that patients in the intervention group were less likely to relapse.

It has been suggested that the reduction in relapse in BD patients after PE for caregivers, may be due to better adherence to medication, timely recognition of manic symptoms, and lowering the intensity of Expressed Emotions (EE) (Honig et al. 1997). In families with low EE aberrant behaviour of the patient is attributed to illness instead of personal shortcomings, resulting in better acceptance and understanding, in turn associated with reducing relapse (Miklowitz 2004; Peris and Miklowitz 2015).

In the Netherlands, PE groups have been part of the regular treatment for patients with BD since the 1990′s (van Gent and Zwart 1991), typically offering six sessions group PE for patients together with one caregiver (Hofman et al. 1992). Over the years, format, frequency and content of PE groups offered across the country had deviated from that original format and from evolving research evidence. Therefore, the Dutch Foundation for Bipolar Disorders (www.kenbis.nl) decided to update and harmonize the PE program by integrating evidence from the existing research literature with clinical and patient experience. A task force including professionals and members of the patient and caregiver advocacy organisation, developed a new 12-sessions group PE program for patients together with their caregivers. As in the original format, it was assumed that the combined attendance of patients and their caregivers in the same group and during all sessions supports mutual understanding and facilitates a triadic collaborative care approach in the subsequent long-term treatment. Expanding from a 6-session to a 12-session format was chosen given the evidence for the efficacy of a 12-session program for patients (Castle et al. 2007; Castle et al. 2010), caregivers (Reinares et al. 2008), and patients and caregivers (D’Souza et al. 2010). Moreover, a 21-session program (Colom et al. 2003a) was considered too lengthy when both patients and caregivers should attend all sessions. The resulting new PE program was then reviewed by an independent committee of the patient and caregiver organisation for BD, which resulted in minor adaptations. For the present study the final PE program was introduced in mental health centers throughout the Netherlands that collaborate in the network of the Dutch Foundation for Bipolar Disorders.

### Goals

The primary goal of this study was to evaluate the feasibility of the 12-sessions PE group program for patients with BD and their caregivers in routine outpatient practice, based on the evaluation of format and content by participants and course leaders, and attendance and dropout. A secondary goal was to explore effectiveness on mood symptoms, attitudes towards BD and its treatment, levels of self-management, and levels of expressed emotion.

## Methods

The study was a multi-center study carried out in outpatient clinics of mental health institutions throughout the Netherlands, specialised in the treatment of BD and participating in the Dutch Foundation for Bipolar Disorders network (www.kenbis.nl). Each participating outpatient clinic organized one new format PE group.

The study was approved by the Institutional Review Board of the VU University Medical Center, Amsterdam. All study participants provided written consent to participate after given full information about the study.

### Subjects

Patients aged 18 years or over with (1) a by the treating psychiatrist confirmed diagnosis of BD-I, BD-II or BD-NOS who were currently in treatment for BD; (2) who had an indication for a group PE as part of their regular treatment as recommended in the Dutch Guideline for BD; (3) were not currently in a manic or major depressive episode as assessed by their treating psychiatrist; and (4) had a good understanding of the Dutch language and the nature of intervention, were asked to participate in this newly designed PE group program, by their mental health care professional, together with one caregiver. Subsequently, all participants of this group PE were invited to participate in the current study evaluating this new program by completing questionnaires at two time points during the program. There were no other in- or exclusion criteria. Those participants (patients and caregivers) who choose to take part in the study gave written informed consent. In addition, a total of 35 psychiatric nurses, psychiatrists, and psychologists conducting the PE groups in the various centers, participated in the study as course leaders, and were asked to share their experience with the program by completing evaluation forms after the last session.

### Intervention

The PE program consisted of 12 weekly group sessions of 2 h, including a 30 min break. The content of the sessions was based on themes common in effective PE programs aiming at improvement of illness awareness, early detection, treatment adherence, and lifestyle adjustments (Table 1). Every PE group had a maximum of 16 participants, typically eight patients and eight related caregivers. Each patient was invited to bring one caregiver. The patients and caregivers received a workbook. Two mental health professionals with experience in BD treatment led the course following a manual describing content and execution of each session and using a slide set for each session. At least one of the course leaders had specific experience with BD PE groups.

**Table 1:** Content of the psychoeducation program

Session number	Theme
1	Meeting other participants and introduction to psychoeducation
2	Introduction to bipolar disorders, manic episodes, misconceptions, and prejudices
3	Depression, cognitive problems, and the role of caregivers
4	Causes, course, heredity, and desire to have children
5	Medication I: considerations about use, types of medication, and lithium
6	Medication II: adherence, monitoring, and other medication than lithium
7	Self-management: Life Chart, Relapse Prevention Plan, and lifestyle
8	Stress management, problem-solving strategies, communication skills, and psychotherapy
9	Relapse Prevention Plan I: depressive episodes and suicidal thoughts
10	Relapse Prevention Plan II: (hypo) mania, mixed episodes, and involuntary admission
11	Psychosocial aspects: the influence of bipolar disorders on relationships and work
12	Closing session, meeting the Dutch patient and caregiver advocacy organization, evaluation, and ‘further on your own’

### Evaluation and measures

*Patient measures* Patient and illness characteristics were concisely assessed through questionnaires after session 1. After session 12, an evaluative questionnaire, including both closed and open-ended questions was administered. This questionnaire was constructed for the purpose of evaluating the various aspects of the PE group program, including content (by session) and form (duration, number of sessions, course materials). Closed questions were evaluated on a semantic differential scale ranging from 1 to 5, with 1 (‘very bad’) and 5 (‘very good’). Through open-ended questions patients were asked (1) what they found had been the most useful to them throughout the program, and (2) which aspects were redundant.

Attendance per session was evaluated on a separate form. Additionally, patients completed several questionnaires at baseline session 1 and final session 12 to evaluate the effect of the intervention on several symptom and behavioural dimensions. The self-rated Quick Inventory of Depressive Symptomatology (QIDS-SR; Rush et al. 2003) was used to assess current depressive symptoms. Manic symptoms were assessed using the Altman Self-Rating Mania Scale (ASRM; Altman et al. 1997). The short form of the Patient Activation Measure (PAM-13; Hibbard et al. 2004; Moljord et al. 2015) was used to measure patients’ self-reported knowledge, skills and confidence for managing their BD. With this instrument the individual patients’ level of activation can be reliably determined; higher activation scores representing better knowledge, skills and confidence. A positive change in activation has been shown to be associated with positive changes in various self-management behaviours, such as self-care and preventive behaviours, in patients with chronic illness. The Level of Expressed Emotion (LEE; Cole and Kazarian 1988) was used as a self-report questionnaire to assess the perception of levels of EE in family interactions in the past month, higher scores indicating increased levels of expressed emotion (EE). A high level of EE is considered to reflect an adverse interaction pattern towards the patient with a mental illness, as perceived by the patient. In addition to providing an overall score, the LEE consists of four subscales ‘Lack of emotional support’, ’Intrusiveness’, ‘Irritability’ and ‘Criticism’. Finally, the Drug Attitude Inventory (DAI-10; Hogan et al. 1983) was administered to assess the self-reported explicit attitudes towards psychotropic medication.

*Caregiver measures* After session 12, similar evaluative questionnaires to evaluate the PE program as in patients, were administered in caregivers. Additionally, at session 1 and 12 the LEE was administered to assess levels of EE as perceived by caregivers. The LEE (Cole and Kazarian 1988) is typically administered to the recipient, but for the purpose of the current study, the questions for caregivers were modified in order to measure self-appraised attitudes of the caregiver towards the patient.

*Course leader measures* After session 12 course leaders were asked to complete an PE evaluation form in order to evaluate the new PE grogram from the perspective of the course instructors.

### Data analyses

The current program was evaluated using qualitative analyses of answers on the evaluation forms. For the answers to closed questions with semantic differential scales, the mean was calculated. Answers to the open questions were analysed through open and axial coding, followed by further selective coding, by two authors (NJ and SZ) independently. The data was then triangulated between the authors. When discordant, the findings were discussed until consensus was reached.

Answers of patients and caregivers were analysed separately to evaluate the impact of the intervention on symptoms, drug attitudes, level of expressed emotions and self-management. We performed paired T-tests to test for significant (p < 0.05) changes between T0 and T1. The data was checked for outliers. Data with more than 5% missing values were excluded from the analysis. A regression analysis was performed to investigate if severity of depression at T0, or total illness years, could predict other significant measures. Statistical analyses were performed using SPSS version 24.

## Results

### Sample

*Centers* In total 17 outpatient clinics of 13 mental health institutions throughout the Netherlands participated in the study, and each clinic organized one PE group.

For demographics of patient and caregivers see Table 2.

**Table 2:** Characteristics of participants of a newly designed 12 session psychoeducational program for bipolar disorder

Patients (n = 97)		Caregivers (n = 72)	
Age (mean) (SD; range)	40.8 (12.6; 19–72)	Age (mean) (SD; range)	50.1 (11.9; 23–72)
Gender (% female)	n = 56 (57.7%)	Gender (% female)	n = 33 (45.8%)
QIDS (mean) (SD; range)	9.0 (6.2; 0–24)		
ASRM (mean) (SD; range)	1.7 (2.5; 0–9)		
Duration of illness in years (SD; range)	13.9 (11.8; 0–45)		
BD diagnosis (self-reported)^a^		Relationship (%)	
BD I (%)	n = 36 (37.1%)	Spouse	n = 48 (66.7%)
BD II (%)	n = 35 (36.1%)	Parent	n = 14 (19.4%)
BD other (%)	n = 4 (4.1%)	Other	n = 8 (11.1%)
Unknown to patient (%)	n = 22 (22.7%)	Not reported	n = 2 (2.8%)
Age at first treatment (SD; range)	32.8 (12.2;12–61)	Years involved in treatment of patient (SD; range)	4.5 (5.9;0–30)
Participating with caregiver (%)	n = 73 (75.3%)		
Participating w/o caregiver (%)	n = 24 (24.7%)		
Currently using medication (%)	n = 88 (90.7%)		
Lithium (%)	n = 54 (61.4%)		
Anticonvulsant ^b^ (%)	n = 28 (31.8%)		
Antipsychotic ^c^ (%)	n = 24 (27.3%)		
Antidepressant (%)	n = 36 (40.9%)		
Anxiolytic (%)	n = 6 (6.8%)		
Number of medications, median (range)	1 (1–4)		

^a^ Patients were asked about their diagnosis; before inclusion in the PE program, all patients had a clinically confirmed diagnosis of bipolar disorder type I, II or NOS

^**b**^ Anticonvulsants included valproate, lamotrigine, carbamazepine

^c^ Antipsychotics were mainly second generation agents

*Patients* A total of 108 BD patients participated in the study of which 101 completed assessments at T0, and 90 at T1. Seven patients, who did not complete the pre-test, completed the evaluative questionnaires at session 12, whereas 18 patients who completed the pre-test did not complete the evaluation forms at the last session. Over three quarter of patients attended the program with their caregiver. The majority of patients used medication for BD. Patients were relatively euthymic at study entrance given low QIDS and ASRM scores. Of the patients attending without a caregiver the mean age was not significantly different from the group attending with a caregiver. There was a difference in sex, as 68% in the group attending without a caregiver were women, compared to 54% in the other group.

*Caregivers* A total of 88 caregivers participated in the program, of whom the majority were the spouses of participating patients (see Table 2). Thirteen caregivers did not complete the pre-test but did complete the last session, whereas 22 caregivers who completed the pre-test did not complete the last evaluative session.

*Course leaders* A total of 35 psychiatric nurses, psychiatrists, and psychologists, typically 2 per PE-group, participated in the study as course leaders. The majority (80%) had over 5 years of experience in the treatment of patients with BD, and in giving a previous PE course for BD.

### Attendance and drop-out

Across study centers the median number of patients per group was 6 (range 3–10), and a median of 5 (range 3–8) caregivers participated per group.

There were 90 (83%) patients and 66 (75%) caregivers who attended the last session of the program. Of the 17 participating outpatient clinics, 12 provided detailed information about drop-out and attendance. This group consisted of 67/108 patients who also accounted for the attendance of the caregivers.

Of the evaluated group the median numbers of sessions attended by the patients was 11, with an average of 10.9 sessions. For the caregivers the median number of sessions attended was 10, with an average of 9.2 sessions.

### Program evaluation

Overall, results of the program evaluation are presented in Table 3. The program was rated as ‘good’ both by patients and their caregivers (mean scores 4.11 and 4.14 respectively), and most course topics were generally rated as ‘good’. The opportunity of (guided) group discussion was rated as very useful. With respect to the format of the course, the length of the sessions was considered ‘good’ by a great majority of the patients and caregivers (84.1% and 84.8% resp.). 71.6% of patients found the number of sessions ‘adequate’ whereas this was considered ‘too many’ by 23.9% of patients. Similar numbers were found for caregivers. The quality of course materials and visual media was rated as ‘good’ by both patients and caregivers (3.90 and 3.95 resp.).

**Table 3:** Program evaluation by patients, caregivers and course leaders(mean scores bases on differential scale ranging from 1 to 5. with 1 (‘very bad’) and 5 (‘very good’)

	BD patients		Caregivers		Course leaders	
	n	Mean (SD)	n	Mean (SD)	n	Mean (SD)
Overall rating	73	4.11 (0.49)	57	4.14 (0.52)	34	4.00 (0.07)
Specific topics:						
Mania and mixed episodes	86	3.84 (0.73)	62	3.98 (0.59)	32	3.97 (0.31)
Depression	87	3.71 (0.82)	64	3.92 (0.63)	32	4.00 (0.36)
Cognitive complaints	85	3.58 (0.70)	64	3.64 (0.74)	–	–
Causes, course	86	3.65 (0.87)	62	3.77 (0.71)	32	3.81 (0.64)
Heredity, desire to have children	84	3.32 (0.92)	58	3.52 (0.78)	32	3.88 (0.49)
Medication and considerations	86	3.84 (0.84)	64	3.86 (0.83)	34	3.91 (0.57)
Self management, lifestyle	85	3.81 (0.75)	64	3.94 (0.77)	32	3.84 (0.68)
Lifechart, relapse prevention plan	85	3.67 (0.81)	62	3.81 (0.94)	31	3.84 (0.58)
Stress management, communication	84	3.64 (0.72)	60	3.70 (0.77)	30	3.77 (0.57)
Psychosocial aspects	84	3.61 (0.73)	60	3.82 (0.73)	30	3.97(0.49)
Evaluation and closing session	77	3.58 (0,85)	55	3.69 (0.77)	28	4.07 (0.38)
Contributing aspects:						
Group discussion	80	3.96 (0.67)	63	4.03 (0.67)	30	3.67 (0.88)
Patient and caregiver advocacy organization	71	3.01 (1.1)	51	3.00 (1.1)		
Homework	82	2.95 (0.80)	57	2.75 (0.85)		
Lifechart	71	3.25 (0.98)	29	2.72 (1.3)		

Also the course leaders, running the newly developed program for the first time, generally rated the program as ‘good’ (see Table 4) with and individual topics rating between 3.06 (last evaluation session) and 3.77 (medication). Course materials were rated as good. And the majority of course leaders (74.3%) found enough opportunity for group discussion. The number of sessions in relation to the course content was rated as ‘good*’* (as opposed to too many or too little) by 68.6% of the course leaders. Of the course leaders, 51.4% considered the number of sessions less convenient, whereas the number of sessions was considered acceptable by 62.9% of the patients and 55.2% of the caregivers.

**Table 4:** Symptom and behavioral outcome measures for patients and caregivers participating in a 12-session psychoeducation group program for bipolar disorder

Patients					
	T0		T1		Paired t-test
QIDS - SR	n = 81	8.60 (6.14)	n = 81	7.40 (5.91)	t = 2.26; p = 0.03
ASRM	n = 82	1.82 (2.60)	n = 82	1.73(2.07)	ns
PAM-13 total	n = 67	53.59 (11.9)	n = 67	56.93 (14.1)	t = − 2.21; p = 0.03
PAM level 1	n = 88	32 (36%)	n = 83	18 (22%)	
PAM level 2		24 (27%)		17 (20%)	
PAM level 3		18 (21%)		24 (29%)	
PAM level 4		14 (16%)		24 (29%)	
LEE Total	n = 73	69.56 (18.7)	n = 73	70.70 (19.5)	ns
Subscales:					
LES (n = 75)		33.72 (9.9)		34.31 (9.6)	ns
INTR (n = 70)		15.77 (6.1)		15.77 (5.7)	
IRR (n = 69)		11.55 (4.3)		11.88 (3.9)	
C (n = 73)		8.88 v (3.3)		8.78 (3.0)	
DAI 10 mean score (1 positive, − 1 negative)	n = 59	1.46 (2.70)	n = 59	1.63 (2.56)	ns
Caregivers					
	T0		T1		
LEE Total	n = 45	70.85 (11.8)	n = 45	67.59 (12.4)	T = 2.31; p = 0.03
Subscales:					
LES (n = 50)		32.66 (8.0)		31.64 (7.8)	
INTR (n = 50)		15.32 (4.9)		14.32 (4.3)	
IRR (n = 48)		11.98 (3.4)		10.90 (3.4)	ns
C (n = 51)		9.16 (2.4)		8.63 (2.2)	

QIDS- SR, self-rated Quick Inventory of Depressive Symptomatology; ASRM, Altman Self-Rating Mania Scale; PAM-13, short form of the Patient Activation Measure; DAI 10, Drug Attitude Inventory; LEE, Level of Expressed Emotion (LES, lack of emotional support; INTR, intrusiveness; IRR, irritability; C, criticism)

Open-ended questions on the evaluation forms asked about the most appreciated aspects of the program, and which topics could be left out. Patients (n = 86) referred most often to the exchange of experiences with other patients and mutual recognition as the most valuable part of the program. They appreciated sharing information and learning how other patients cope with their illness. Patients also reported that the program resulted in heightened self-awareness and self-reflection. In addition, more informative aspects were mentioned, learning self-management skills, early warning signs, and knowledge about the illness and drug treatment. The caregivers (n = 61), similarly to the patients, answered that they found sharing and exchange of information most valuable. Also, knowledge about the illness was mentioned, and the fact that the program contributed to increased understanding of their family member with BD.

Of the patients (n = 64), the majority answered that nothing should be left out. However, some mentioned that the two sessions on medication could be more concise. Caregivers (n = 34) most often answered that nothing should be left out and added that theoretical information regarding biology and medication was sometimes too complex.

### Treatment effect analyses

Results of scores symptom and behavioural dimensions before and after the PE program are presented in Table 4.

For patients, severity of depressive symptoms as measured by QIDS, was significantly decreased after the PE program (p = 0.03) and the PAM-13 activation score indicated a significant increase in self-management skills at T1 compared to T0 (p = 0.03). Manic symptomatology, perceived EE, and the attitude towards psychiatric medication did not differ between T0 and T1. We additionally tested models for prediction of the outcome measures that were significantly different between T0 and T1 (QIDS and PAM-13), with as predictor either the QIDS score at baseline or the total years of illness. We found no significant predictors, except for the difference in QIDS score, for which the QIDS score at T0 significantly predicted the change at T1 (*β* = − 0.43, *p* < 0.001): indicating that those having more depressive symptomatology at T0 show less decrease in depressive symptoms at T1.

Although perceived EE did not differ pre- and post-treatment in BD patients, the LEE total score in caregivers was significantly lower after the PE group program (p = 0.03), indicating less self-reported expressed emotions towards the patient by the caregiver. Further inspection of the subscales using a repeated multivariate measure analysis including the subscales gave no significant overall effect. This may be due to a lack of power when analysing subscales. Exploration of subscales suggested that subscales intrusiveness (INTR) and perceived irritation (IRR) are to account for the effect.

## Discussion

This study investigated the feasibility of a newly designed 12-session group psychoeducation program for patients with BD and their caregivers, and explored its effectiveness in a non-controlled, naturalistic multi-center treatment setting. A high percentage of both patients (83%) and caregivers (75%) completed the program with a high average attendance rate. Both the content and practicability of the program were rated as good by both participants and course leaders. Qualitative analyses of subjective experiences demonstrated that the reciprocal sharing of information was rated as particularly valuable by patients as well as their caregivers. In addition, the patients reported to have gained a heightened self-awareness and increased self-management skills. The number of sessions as well as the program length was well tolerated as shown by a high rate of attendance by patients and caregivers. This is critical since both patients and caregivers attend all 12 weekly sessions that are mostly held in the late afternoon, i.e. during working hours. This was a major consideration to design a 12-sessions rather than a 21-sessions program, but still twice as long as the original 6-session PE group program that had been predominantly used in our country for two decades.

Although the naturalistic design of our study prevents firm conclusions, exploring the effectiveness of this PE program showed a significant decrease in the severity of depressive symptoms and an increased level of self-management skills in patients. Empowering patients to take a prominent role in their own health issues is the core of every PE program and has been related to fewer hospitalizations, a better medication adherence, and an improved physical and mental status throughout many different chronic somatic and psychiatric conditions (Schulman-Green et al. 2012; Kukla et al. 2013; Levitt et al. 2009).

Caregivers reported significantly lower expressed emotion (EE) towards the patient post-treatment, and perceived themselves as being less in need of controlling and monitoring the patient, including a less stressful coping with the patient. This could be the result of a better understanding of the illness and more confidence in the patient’s self-management skills. This is of importance since low EE contributes to preventing relapse (Miklowitz 2004). In contrast, patients did not perceive a lower EE after treatment, indicating some incongruence in perception between patients and their caregivers. A longer follow-up may have revealed delayed effects through changed behaviours.

Designed primarily to investigate feasibility and acceptability in daily clinical practice, our study has several limitations. Since we did not include a control group, the results regarding short-term effects are only indicative, and given the lack of follow-up assessments after the intervention it remains unknown whether there is an effect on recurrence rates, hospitalisations, or medication adherence. A future study including a control group and follow-up measurements is needed to distinguish the short- and long-term effects of this PE program from the naturalistic course of illness under treatment as usual. In addition, it may be of interest to investigate not only possible moderators, but also the mediators of the effect of PE such as influence of stage of the disorder (Berk et al. 2017), the severity of current depressive and manic symptoms, and the influence of residual cognitive impairments.

Despite these limitations, we consider the results of our study as highly generalizable, since it was implemented in daily clinical practice in a large number of mental health centers, and offered to all patients and their caregivers who had not yet attended a PE program at that time. Since all materials (manual for participants and manual for course leaders) are distributed by the office of the Dutch Foundation for Bipolar Disorders (www.kenbis.nl), we have an indication of the implementation after this study was completed. In 2018 and 2019, in total 1808 manuals for participants and 156 manuals for course leaders were sent to 37 different mental health institution in the Netherlands. These numbers do not include the manuals that were previously distributed for the current study. Since manuals were ordered repeatedly by the same institution, this suggests that this PE course has become part of routine practice in these centers.

## Conclusion

Targeted at both patients and caregivers, this compact 12-sessions psychoeducation group program showed good feasibility and was well accepted by participants and course leaders. Preliminary effects on measures of self-management, expressed emotions, and depressive symptoms were promising. After its introduction it has been widely implemented in mental health institutions throughout the Netherlands.

## Data Availability

The datasets used and/or analyzed during the current study are available from the corresponding author on reasonable request.

## Abbreviations

BD: Bipolar disorder
EE: Expressed emotion
PE: Psycho-education
QIDS: Quick Inventory of Depressive Symptomatology (self-rated)
ASRM: Altman Self-Rating Mania Scale
PAM-13: Patient Activation Measure
DAI-10: Drug Attitude Inventory
LEE: Level of expressed emotion
